# Computer-Guided Intraosseous Anesthesia as a Primary Anesthetic Technique in Oral Surgery and Dental Implantology—A Pilot Study

**DOI:** 10.3390/dj13120572

**Published:** 2025-12-03

**Authors:** Minou Hélène Nilius, Manfred Nilius

**Affiliations:** 1 Niliusklinik, Londoner Bogen 6, 44269 Dortmund, Germany; minou@niliusklinik.de; 2 School of Dentistry, Goethe University Frankfurt, 60596 Frankfurt am Main, Germany; 3 Department of Oral and Maxillofacial Surgery, University Hospital “Carl Gustav Carus”, Technische Universität Dresden, 01307 Dresden, Germany

**Keywords:** anesthesia, dental, anesthetics, local, nerve block, mandibular nerve, pain perception, dental implantation, endosseous, oral surgery, articaine, adverse effects, maxillary nerve block

## Abstract

This pilot study evaluated the feasibility and preliminary outcomes of computer-guided intraosseous anesthesia for oral surgery and dental implantology. **Background/Objectives**: The inferior alveolar nerve block (IANB) is widely used for dental anesthesia; however, issues such as anatomical variation and inflammation can hinder effective pain control. Alternatives have been studied primarily in irreversible pulpitis, with limited data available for other procedures. **Methods**: In a retrospective analysis, data from 85 patients who underwent implantation, root resection, or osteotomy using QuickSleeper^®^ intraosseous anesthesia (IO), infiltration (INF), or IANB were assessed. **Results**: IO, IANB, and INF produced similar pain levels during administration, procedure, and recovery; blood pressure and heart rate were comparable. IO and INF led to less lip numbness after 15 min and required less anesthetic. IO had a significantly shorter latency than IANB, allowing earlier surgery. **Conclusions**: Computer-guided IO is a viable alternative to IANB for implantation, root resection, and osteotomy, offering equal pain control, shorter latency, earlier surgery, and reduced injection volume. Within the limitations of this pilot study, the findings should be considered preliminary and require confirmation in larger prospective studies. Given the exploratory pilot design, no formal sample size calculation was performed; the sample size was defined by feasibility considerations.

## 1. Introduction

Local anesthesia is essential in modern dentistry, providing pain-free diagnostic and therapeutic procedures. Effective anesthesia improves patient comfort, cooperation, and treatment outcomes [1]. However, conventional inferior alveolar nerve block (IANB), or in the maxilla (maxillary nerve block, MNB), can be challenging due to anatomical variability, inflammation, patient anxiety, or the operator’s experience [2]. Side effects include pain at the injection site, swelling, limited mouth opening, soft tissue injuries, and prolonged numbness [3]. To address these challenges, alternative anesthesia techniques have been developed, such as buccal infiltration, Gow-Gates, closed-mouth nerve block, INF, intrapulpal injection, and intraosseous anesthesia (IO). Computer-controlled rotary injection syringes facilitate IO delivery. Previously, we compared intraosseous anesthesia [IO] [4] with conventional methods in patients with irreversible pulpitis [5]. Herein, we compare IANB/MNB, INF, and IO using a computer-controlled rotary injection syringe in patients undergoing implantation, root resection, and osteotomy.

## 2. Materials and Methods

### 2.1. Study Design

As a pilot study, the investigation was conducted with a limited convenience sample; no a priori power or sample size calculation was performed.

This study was designed as a retrospective study using a convenience sample of consecutive patients in our practice. This study was authorized by the Committee for Ethics, Dresden University of Technology, Carl Gustav Carus, Case Ref. no: BO-EK-389082022/2022-09-15. All patients were informed thoroughly about the study and provided written informed consent. The study was conducted in accordance with the principles outlined in the Declaration of Helsinki.

Patients were seen between February 2022 and February 2024 at our dental clinic in Germany for implantation, root resection, and osteotomy procedures. A total of 85 patients were included in the study. The average age of the patients was 52.6 ± 19.9 years. 27 (31.8%) were male, the remainder were female (n = 58, 68.2%). 12 patients (14.1%) used nicotine products, and 16 (18.8%) had preexisting cardiovascular disease. Distal teeth were present in 67 patients (78.8%).

The patients were undergoing implantation, root resection, or osteotomy, with some patients undergoing multiple procedures on different dates. Local anesthesia methods were chosen for each procedure by the treating dentist, in accordance with clinical considerations (IANB, INF, IO). Computer-guided IO was carried out using a computer-controlled rotary injection syringe (QuickSleeper5, Orcos Medical, Küsnacht, Switzerland) according to the manufacturer’s instructions. For all procedures, we used 1.7 mL of articaine hydrochloride with 1:100,000 epinephrine hydrochloride (Ultracain D-S, Sanofi-Aventis, Frankfurt, Germany). Table 1 shows the number of procedures and anesthesia methods employed. The location of the procedure in the lower or upper jaw is shown in Table 2.

### 2.2. Statistics

All analyses are exploratory and descriptive in nature, given the pilot design; results should be interpreted cautiously.

Statistical analysis was performed using the SPSS Statistics Version 31.0.1.0 (IBM, Armonk, NY, USA). Differences in categorical data were tested for significant difference using the Chi-square test, while continuous data were tested using the paired *T*-test. Due to the relatively small sample size, no corrections were made for multiple testing or possible confounding parameters such as comorbidities and differences in the demographic background of the patients. The effectiveness of the three anesthetic techniques—computer-guided intraosseous anesthesia (IO), inferior alveolar nerve block (IANB), and infiltration anesthesia (INF)—was assessed based on several objective clinical outcome parameters rather than on subjective patient perception alone. For each procedure, the latency time to the onset of anesthesia, defined as the interval between the administration of the local anesthetic and the beginning of surgery, was recorded as an indicator of functional anesthetic efficiency. The total injection volume required to achieve sufficient anesthesia and the necessity for additional injections were likewise documented to evaluate clinical adequacy. The presence or absence of soft-tissue numbness at 15 min after injection served as a clinical sign of anesthetic diffusion, independent of patient reporting. Moreover, systolic and diastolic blood pressure and heart rate were monitored intraoperatively as physiological markers of discomfort or nociceptive activation. The readiness for incision, determined by the operator, reflected the practical onset and workflow efficiency of each anesthetic technique. In addition to these parameters, patient-reported pain was recorded using a standardized 10-point visual analogue scale (VAS) at multiple timepoints; however, the overall assessment of anesthetic effectiveness relied primarily on these objective, operator-observed, and physiological measures rather than on subjective evaluations alone.

## 3. Results

In all procedures, one or two perforations were required. Fewer patients in the IO and INF groups needed two perforations than in the IANB group; this difference reached statistical significance only in the comparison between IANB and INF (IO vs. IANB/MNB, *p* = 0.0744; INF vs. IANB/MNB, *p* = 0.0021, chi-square test). There was no significant difference in perforation between IO and INF (Table 3, *p* = 0.0841).

Needle obstruction was infrequent and occurred at similar rates for all anesthesia methods (Table 4, *p* > 0.05).

Needle changes were rare across all procedures and showed no significant difference between anesthesia methods (Table 5).

Lip numbness was common with IANB but rare with IO or INF at 15 min post-injection (Table 6). Differences between IANB and both IO and INF were statistically significant (*p* < 0.0001).

Lip numbness rates were similar for IO and INF (*p* = 0.1706) in the mandible. Additional injections were needed in 14.0% to 21.6% of procedures, with no significant differences between anesthetic methods (Table 7, *p* > 0.005).

**Figure 1:**
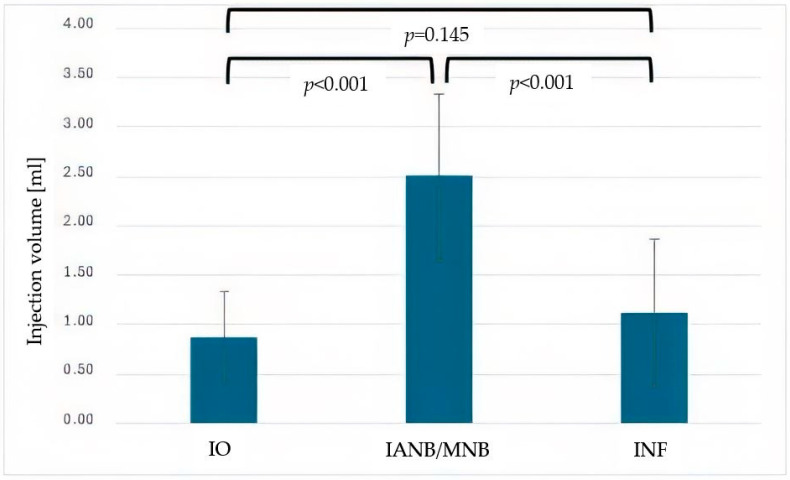
Mean injection volume (mL) for intraosseous anesthesia (IO), inferior alveolar nerve block (IANB), maxillary nerve block (MNB) and infiltration anesthesia (INF). Error bars represent standard deviation (StD). Significant differences between IANB and both IO and INF are indicated (*p* < 0.001).

**Figure 2:**
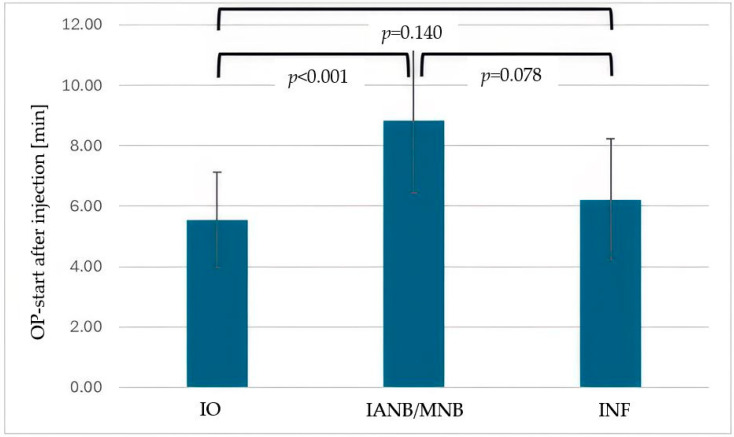
Mean time (minutes) from anesthesia administration to the start of surgery for intraosseous anesthesia (IO), inferior alveolar nerve block (IANB), maxillary nerve block (MNB) and infiltration anesthesia (INF). Error bars represent standard deviation (StD). IO enabled significantly earlier procedure start compared with IANB (*p* < 0.001).

The IANB/MNB group received a significantly larger injection volume than the IO or INF groups (Figure 1, *p* < 0.001), with no volume difference between the IO and INF groups (*p* = 0.145). IO enabled a significantly earlier start compared to IANB/MNB (Figure 2, *p* < 0.001), and showed no significant timing difference compared to INF (*p* = 0.140).

**Figure 3:**
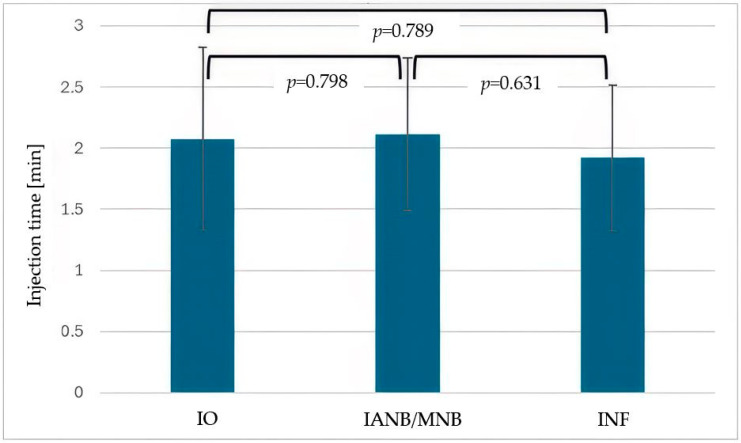
Mean injection time (minutes) for intraosseous anesthesia (IO), inferior alveolar nerve block (IANB), maxillary nerve block (MNB) and infiltration anesthesia (INF). Error bars represent standard deviation (StD). No statistically significant differences were observed between the three techniques.

**Figure 4:**
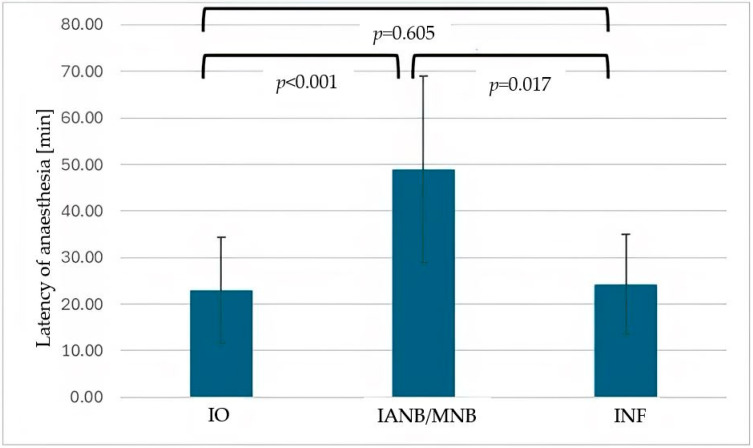
Mean latency of anesthesia onset (minutes) for intraosseous anesthesia (IO), inferior alveolar nerve block (IANB), and infiltration anesthesia (INF). Error bars represent standard deviation. Latency was significantly longer for IANB compared with IO (*p* < 0.001) and INF (*p* = 0.017).

The difference in the start of the procedure after injection, when comparing IANB/MNB and INF, was also not statistically significant (*p* = 0.078). The injection time was the same for all three local anesthesia methods (Figure 3, *p* > 0.005, respectively). The latency of anesthesia was longest for IANB/MNB, with *p*-values of *p* < 0.001 for the comparison between IO and IANB/MNB and *p* = 0.017 for IANB/MNB and INF (Figure 4). There was no significant difference in the latency of anesthesia between IO and INF (*p* = 0.605).

Patient-reported pain, measured using a 10-point visual analogue scale, was compared for pain during infiltration, perforation, instillation, treatment, post-surgery, and one day post-surgery. There was no significant difference in these pain levels between IO, IANB/MNB, and INF (Table 8).

Other patient stress level-associated parameters that were compared between IO, IANB/MNB, and INF included systolic and diastolic blood pressure, as well as heart rate. The values were similar for all three anesthesia types with no significant differences (Table 9).

## 4. Discussion

Local anesthesia is an essential part of dentistry, with IANB being the most commonly used method for routine procedures such as fillings, root canals, implantations, and extractions [6]. However, due to various factors, reaching sufficient depths of anesthesia using IANB can be challenging in some patients, and alternative anesthesia methods have been investigated [4]. Typically, the success rate of IANB is around 70–90%. However, inflammation in the injection area and pulpitis severely reduce its efficacy [7,8,9,10,11].

Comparisons of the anesthetic efficacy of IANB with other local anesthesia methods have been published [12,13,14]. However, published comparisons between IANB and IO, including a study conducted by our group, are limited to the treatment of irreversible symptomatic pulpitis, a condition known for the lack of efficacy of IANB [5,15,16]. Here, we compared IO, IANB/MNB, and INF as anesthesia methods for implantation, root resection, and osteotomy procedures. All three anesthesia methods were comparable with respect to the number of perforations, needle obstructions, and the necessity of a needle change or additional injections, with only INF showing a slight, but statistically significant, lower average number of perforations.

Soft tissue numbness is a common side effect of local anesthesia in dental procedures and can lead to soft tissue injury due to bites or burns, especially in children [17,18]. In our comparison, lip numbness at 15 min was significantly more common in IANB. Local anesthesia with a lower incidence or shorter duration of lip numbness might be a better choice for vulnerable populations such as children or people with developmental disabilities. The result might therefore indicate that both IO and INF might be better suited for these populations. A smaller injection volume used in both INF and IO compared to IANB/MNB might further contribute to its better suitability for vulnerable populations. However, the ultimate test for any anesthetic is the patient’s perception of pain [19,20,21,22,23].

Our results for pain control during treatment and after the surgery showed comparable results for all three methods [8,12,14,15,16,17,24,25,26]. Other researchers have reported better efficacy for IO compared to IANB in the past [16]. However, in the study by Farhad et al., 2018 [16] the anesthetic was used on patients with irreversible pulpitis, a condition that is notorious for being associated with lower efficacy of IANB, which might explain the better efficacy of IO in terms of pain control in that study [8,12,14,15,16,17,18,19,20,21]. Pain during anesthesia administration was also comparable among the three methods, with a slightly lower average number for IO. However, there was a relatively high inter-patient variability, which might have contributed to the fact that significance levels were not reached. Pain perception during dental procedures typically shows a wide variability. This is due to genetic factors, the presence of inflammation, anatomical differences, and psychological factors such as dental procedure anxiety, sensitization from previous procedures, and pain expectation [23,24,25,26]. Future studies should therefore aim for a larger sample size. A prospective study design with stringent inclusion and exclusion criteria could help select a more homogenous study population, potentially making comparisons in pain perception easier.

Limitations of the study: the study employed a retrospective study design with a convenience sample, which could potentially lead to a selection bias. In accordance with the sampling method, the anesthesia method was selected based on operator preference and patient input. This also could lead to potential selection bias. The study had a relatively small sample size of 85 patients and a total of 146 procedures, making it impossible to stratify the data by procedure type. Pain control, i.e., anesthesia effectiveness, was evaluated by subjective patient-reported pain scores rather than an objective pain assessment. Pain levels can be influenced by anxiety, but anxiety levels were not assessed.

This work was conceived as a pilot study with a limited sample size and absence of stratification; larger stratified prospective trials are warranted.

## 5. Conclusions

These preliminary results warrant confirmation in larger, stratified, prospective studies.

Within the limitations of this pilot study, other measures indicating increased patient stress, such as systolic and diastolic blood pressure and heart rate, were also comparable among IO, IANB/MNB, and INF. This indicates that the efficacy of these anesthetic methods was similar when treating patients undergoing implantation, root extraction, or osteotomy. Shorter overall procedures might be perceived as more comfortable by the patients. Therefore, an anesthesia method with shorter latency and shorter waiting time to start surgery might be a better option when other parameters are similar. IO had the shortest latency and earliest start of surgery while displaying similar efficacy, injection time, and pain during anesthesia administration.

Our study using computer-assisted IO, IANB/MNB, and INF as local anesthesia for implantation, root extraction, and osteotomy yielded similar pain control and patient stress levels for all three methods. The injection volume was lower for IO and INF compared to IANB, with shorter latency and earlier surgery start times, making IO a viable alternative to standard IANB/MNB, with the potential benefit of shorter procedure times.

## Figures and Tables

**Table 1:** Surgical procedures and anesthesia methods used in 85 patients.

Surgical Procedure	IO N = 66 (%)	IANB/MNB N = 43 (%)	INF N = 37 (%)	Total N = 85 (%)
Osteotomy	28 (42.4)	20 (46.5)	17 (45.9)	65 (76.5)
Implantation	29 (43.9)	20 (46.5)	12 (32.4)	61 (71.8)
Root resection	9 (13.6)	3 (7.0)	8 (21.6)	20 (23.5)

Distribution of surgical procedures (osteotomy, implantation, and root resection) according to anesthesia method used: intraosseous anesthesia (IO), inferior alveolar nerve block (IANB), maxillary nerve block (MNB) or infiltration anesthesia (INF).

**Table 2:** Location of surgical procedures in the upper or lower jaw.

Location	IO N = 66 (%)	IANB/MNB N = 43 (%)	INF N = 37 (%)	Total N = 85 (%)
Maxilla	29 (43.9)	5 (11.6)	29 (78.4)	65 (76.5)
Mandible	37 (56.1)	38 (88.4)	8 (21.6)	61 (71.8)

Location of surgical procedures in the maxilla or mandible stratified by anesthesia method: intraosseous anesthesia (IO), inferior alveolar nerve block (IANB), maxillary nerve block (MNB or infiltration anesthesia (INF).

**Table 3:** Number of perforations for the main procedure according to anesthesia method.

Number of Perforations	IO N = 66 (%)	IANB/MNB N = 43 (%)	INF N = 37 (%)
1	45 (68.2)	22 (51.2)	31 (83.8)
2	21 (31.8)	21 (48.8)	6 (16.2)

Number of perforations required for the main procedure, comparing intraosseous anesthesia (IO), inferior alveolar nerve block (IANB), and infiltration anesthesia (INF).

**Table 4:** Occurrence of needle obstruction.

Needle Obstruction	IO N = 66 (%)	IANB/MNB N = 43 (%)	INF N = 37 (%)
No	57 (86.4)	40 (93.0)	33 (89.2)
Yes	9 (13.6)	3 (3.5)	4 (10.8)

Occurrence of needle obstruction during intraosseous anesthesia (IO), inferior alveolar nerve block (IANB), maxillary nerve block (MNB) and infiltration anesthesia (INF).

**Table 5:** Necessity of needle change.

Needle Change	IO N = 66 (%)	IANB/MNB N = 43 (%)	INF N = 37 (%)
No	58 (87.9)	41 (95.3)	34 (91.9)
Yes	8 (12.1)	2 (2.4)	3 (8.1)

Necessity of needle change during intraosseous anesthesia (IO), inferior alveolar nerve block (IANB), maxillary nerve block (MNB) and infiltration anesthesia (INF).

**Table 6:** Frequency of lip numbness 15 min after injection.

Lip Numbness	IO N = 66 (%)	IANB N = 43 (%)	INF N = 37 (%)
No	57 (86.4)	9 (20.9)	28 (75.7)
Yes	9 (13.6)	34 (79.1)	9 (24.3)

Frequency of lip numbness 15 min after injection with intraosseous anesthesia (IO), inferior alveolar nerve block (IANB), and infiltration anesthesia (INF).

**Table 7:** Frequency of additional injections required.

Additional Injections	IO N = 66 (%)	IANB N = 43 (%)	INF N = 37 (%)
No	55 (83.3)	37 (86.0)	29 (78.4)
Yes	11 (16.7)	6 (14.0)	8 (21.6)

Frequency of additional injections required during intraosseous anesthesia (IO), inferior alveolar nerve block (IANB), and infiltration anesthesia (INF).

**Table 8:** Average patient-reported pain scores (10-point visual analogue scale).

Pain	IO Mean (StD) N = 66 (%)	IANB/MNB Mean (StD) N = 43 (%)	INF Mean (StD) N = 37 (%)	*p*-Value	*p*-Value	*p*-Value
Infiltration	2.19 (0.69)	2.43 (0.77)	2.33 (1.02)	0.661	0.452	0.592
Perforation	2.41 (0.76)	3.49 (7.01)	4.52 (9.32)	0.336	0.274	0.974
Instillation	2.49 (0.65)	2.49 (0.65)	2.67 (0.84)	1.0	0.135	0.421
Treatment	1.11 (0.31)	1.13 (0.06)	1.11 (0.22)	0.324	1.0	0.98
Post surgery	1.41 (0.50)	1.46 (0.51)	1.19 (0.40)	0.160	0.162	0.96
1-day post-surgery	1.57 (0.50)	1.62 (0.08)	1.57 (0.51)	0.161	1.0	0.331

Average patient-reported pain scores (10-point visual analogue scale) during infiltration, perforation, instillation, treatment, post-surgery, and one day post-surgery for intraosseous anesthesia (IO), inferior alveolar nerve block (IANB), and infiltration anesthesia (INF).

**Table 9:** Average systolic/diastolic blood pressure and heart rate.

Pain	IO Mean (StD) N = 66 (%)	IANB/MNB Mean (StD) N = 43 (%)	INF Mean (StD) N = 37 (%)	*p*-Value	*p*-Value	*p*-Value
(RR sys mmHg)	141 (18)	143 (19)	142 (14)	0.298	0.558	0.400
(RR dia mmHG)	76 (10)	74 (8)	74. (4)	0.647	0.307	1.0
Heart rate (bpm)	80 (21)	84 (22)	81 (9)	0.878	0.934	0.799

Average systolic (RR sys) and diastolic blood pressure (RR dia) in mmHg and heart rate (bpm) measured during procedures using intraosseous anesthesia (IO), inferior alveolar nerve block (IANB), and infiltration anesthesia (INF).

## Data Availability

The original contributions presented in this study are included in the article. Further inquiries can be directed to the corresponding author.

## Abbreviations

bpm	beats per minute
IANB	inferior alveolar nerve block
INF	infiltration anesthesia
IO	computer-guided intraosseous anesthesia
RR dia	diastolic blood pressure (mmHg)
RR sys	systolic blood pressure (mmHg)
SPSS	Statistical Package for the Social Sciences
StD	standard deviation
